# Lack of diversity at innate immunity Toll-like receptor genes in the Critically Endangered White-winged Flufftail (*Sarothrura ayresi*)

**DOI:** 10.1038/srep36757

**Published:** 2016-11-09

**Authors:** Desire L. Dalton, Elaine Vermaak, Hanneline A. Smit-Robinson, Antoinette Kotze

**Affiliations:** 1 National Zoological Gardens of South Africa, P.O. Box 754, Pretoria, 0001, South Africa; 2 Genetics Department, University of the Free State, P.O. Box 339, Bloemfontein, 9300, South Africa; 3 BirdLife South Africa, Private Bag X5000 Parklands 2121, Gauteng, South Africa; 4 Applied Behavioural Ecological & Ecosystem Research Unit (ABEERU), UNISA, Private Bag X6, Florida, 1717, South Africa

## Abstract

The White-winged Flufftail (*Sarothrura ayresi*) population is listed as globally Critically Endangered. White-winged Flufftails are only known to occur, with any regularity, in the high-altitude wetlands of South Africa and Ethiopia. Threats to the species include the limited number of suitable breeding sites in Ethiopia and severe habitat degradation and loss both in Ethiopia and South Africa. Toll-like receptors (TLRs) are increasingly being studied in a variety of taxa as a broader approach to determine functional genetic diversity. In this study, we confirm low genetic diversity in the innate immune regions of the White-winged Flufftail similar to that observed in other bird species that have undergone population bottlenecks. Low TLR diversity in White-winged Flufftail indicates that this species is more likely to be threatened by changes to the environment that would potentially expose the species to new diseases. Thus, conservation efforts should be directed towards maintaining pristine habitat for White-winged Flufftail in its current distribution range. To date, no studies on immunogenetic variation in White-winged Flufftail have been conducted and to our knowledge, this is the first study of TLR genetic diversity in a critically endangered species.

A major focus of conservation genetics is the evolutionary consequences of loss of genetic diversity in a population. Genetic variation can be lost due to population bottlenecks and inbreeding. Loss of heterozygosity can result in an increased probability of homozygosity of deleterious recessive alleles resulting in a limited potential for the species to adapt to changing environments^1^. Currently, several studies report on loss of heterozygosity in wildlife populations based on neutral loci such as microsatellites^2^,^3^, however, their utility in this regard has been debated^4^,^5^. Although these markers are useful for determining migration as well as identifying management units they may not be relevant in studying processes affecting functional diversity. Increasingly, studies are being conducted that analyse the genetic diversity at immune loci to complement research conducted based on neutral markers. Conflicting results have been reported. Oliver and Piertney^6^ indicated a smaller loss of functional diversity in comparison to neutral diversity, whereas Eimes *et al.*^3^ identified that loss of diversity was higher for functional loci. However, populations with few individuals have been reported to have reduced heterozygosity, allelic richness and low variation at functional loci^7^,^8^,^9^,^10^,^11^.

High genetic diversity at immune loci is necessary in order for a population as a whole to be resistant against infectious disease. Immune genes are considered to be the most polymorphic regions in the genome due to adaptive evolution^12^,^13^,^14^,^15^. Diversity in the Toll-like receptor (TLR) genes has been conducted on an increasing number of wild species^16^,^17^. Studies, in both animal and plant genomes, have identified that TLRs are widespread, which may indicate that they play a role as a very ancient recognition system for pathogens^18^. TLRs play a crucial role in the host’s defense against a wide variety of pathogens, including bacteria, viruses and fungi^19^. In mammals, 13 TLRs have been identified to date, while analysis of zebra finch and chicken genomes have identified a total of ten avian TLRs^20^,^21^. Directional selection of TLRs has been observed and provides support for the role of these genes in rapid adaptation to the exposure of new pathogens in new environments^22^. In addition, variability in TLR genes has been reported to be associated with resilience to infections^23^. Higher variation reflects a higher potential for binding a wide variety of pathogens and thus may result in an enhanced ability of the species to adapt to future changes in the environment^24^.

Thus far, several studies have focused on immune diversity at TLRs using model and abundant species^24^,^25^,^26^, whereas analysis on populations with reduced genetic variation is limited^27^,^28^. Here, we examine the influence of a severe population reduction on adaptive (TLR) diversity in the Critically Endangered White-winged Flufftail (*S. ayresi*) populations as well as in one of the most common and widespread Flufftail species, the Red-chested Flufftail (*S. rufa*) for comparison. As TLRs play a key role in host defense, loss of diversity at these functional loci may compromise population survival in this species. The Red-chested Flufftail is currently listed as Least Concern as they are reported to have an extremely large range in Africa^29^. The most threatened Flufftail species, the White-winged Flufftail (*S. ayresi*), is known to occur at 15 sites in South Africa and nearly 4 000 km away at three sites in Ethiopia^30^,^31^. Whether or not the birds migrate between Ethiopia and South Africa has long been an enigma. The AEWA White-winged Flufftail International Single Species Action Plan (ISSAP), created in 2008, emphasizes the limited knowledge on the movements of the birds (whether these are intra-African migrants or altitudinal migrants), which can be an indirect threat to species survival. It is known that the birds occur in Ethiopia between July and September (boreal summer), and in South Africa from November to March (austral summer). The estimated global population size of White-winged Flufftail is less than 250 adults and the species has been listed as globally Critically Endangered. The South African population is estimated to be less than 50 birds^32^. This species, with highly specialized habitat requirements, has a highly-fragmented distribution and is threatened by the loss of the wetland habitat both in Ethiopia and South Africa^33^. Loss of wetland habitat in South Africa is due to mining activities, pollution, crop farming, afforestation, grazing, water abstraction, erosion, peat fires and the development of roads, dams and buildings^34^, whilst in Ethiopia the habitat is mostly threatened by overgrazing and grass-cutting^35^.

In the current study, we apply molecular techniques to quantify TLR genetic diversity in the Critically Endangered White-winged Flufftail by specifically examining diversity in South African and Ethiopian White-winged Flufftail (*S. ayresi*) populations. In addition, TLR diversity was analysed in the more common Red-chested Flufftail (*S. rufa*) species, for comparison. We hypothesize that TLR gene diversity in White-winged Flufftail populations will be similar to those reported for bottlenecked avian populations and lower than more widespread and common species such as the Red-chested Flufftail (*S. rufa*) from South Africa. We are particularity interested in SNPs that are non-synonymous, resulting in functional changes. To our knowledge, this is the first study of TLR genetic diversity in a critically endangered species.

## Results

### Identification of Toll-like Receptors in Flufftail populations

Our primers amplified six TLRs namely; TLR1LA, TLR1LB, TLR3, TLR4, TLR5 and TLR7. The primers tested in this study failed to amplify TLR2, TLR15 and TLR21. Amplification for the six TLRs was successful for all samples included in this study namely; three Red-chested Flufftail, seven White-winged Flufftail from Ethiopia and three White-winged Flufftail from South Africa. For all successful DNA sequences, stop codons, as well as frameshift alterations, were not identified for TLR1LA, TLR1LB, TLR3, TLR4 and TLR7, indicating the absence of pseudogenes. Premature stop codons were, however, observed within TLR5, suggesting the amplification of a TLR5 pseudogene. Thus, subsequent analyses of TLR5 were consequently omitted. Sequence lengths of the analyzed TLRs ranged from 631–1 237 bp. Overall we detected 15 SNPs across the White-winged Flufftail dataset and 27 SNPs in the Red-chested Flufftail population (Table 1). All SNPs were verified in separate, duplicate analyses (PCR and sequencing analysis). In White-winged Flufftail from Ethiopia and South Africa, all loci were polymorphic except for TLR4. In TLR1LA and TLR1LB, a total of five alterations was observed, which included four non-synonymous and one synonymous alteration for TLR1LA. Within TLR1LB, two non-synonymous and three synonymous alterations were detected. TLR7 consisted of four alterations, which included two synonymous and two non-synonymous SNPs. In TLR3 only one synonymous SNP was observed. In Red-chested Flufftail all loci were polymorphic (Table 1). In TLR1LA, five alterations were observed, of which one was non-synonymous. TLR1LB and TLR7 both displayed four synonymous SNPs and no non-synonymous SNPs. TLR3 displayed the highest number of SNPs (N = 10), of which four were synonymous, and in TLR4 an equal number of non-synonymous and synonymous alterations were observed (N = 2). TLR1LA and TLR1LB were the most variable in White-winged Flufftail, whereas in Red-chested Flufftail, TLR3, followed by TLR1LA and TLR7 were the most variable. This is in line with a previous study in which the authors reported that TLR1LA has the highest rate of polymorphism in New Zealand bird species^28^. Significant deviations from Hardy-Weinberg expectations, following Bonferroni correction^36^, were not observed for the majority of loci examined, however a significant heterozygous excess (P < 0.001) was observed for one loci (TLR1LA) in the White-winged Flufftail population from Ethiopia. An absence of positively selected sites was observed within the White-winged Flufftail or Red-chested Flufftail TLRs based on SLAC and REL analysis (Table 2). Two negative/purifying selected sites (codon 190 and codon 220) were, however, identified within the TLR7 gene of the White-winged Flufftail using the REL model. One of these sites (codon 190) was confirmed by the SLAC analyses (Table 2). As with other avian species^16^,^25^, an excess of synonymous substitutions over non-synonymous alterations was observed in both Red-chested Flufftail and White-winged Flufftail. The number of synonymous alterations exceeded the number of non-synonymous alterations in the case of TLR1LB, TLR3 and TLR7, whereas the opposite was observed in the case of TLR1LA. Locations of synonymous and non-synonymous alterations are shown in supplementary material (Supplementary S1). A significant single recombination breakpoint was observed within the TLR7 gene of the White-winged Flufftail, whereas no evidence of recombination was observed in the other TLR genes of the White-winged Flufftail or in any of the TLR genes of the Red-chested Flufftail.

### Differentiation of species and populations

**Figure 1:**
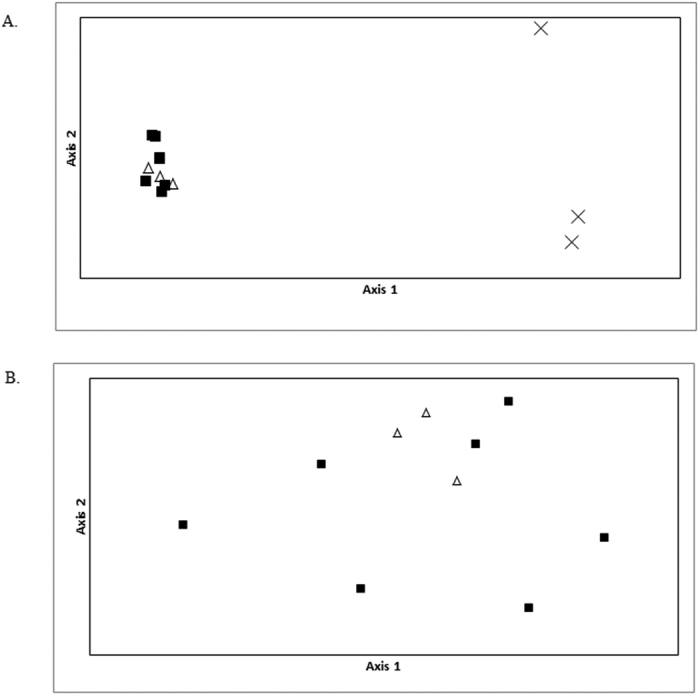
Principal coordinate analysis of five Toll-like receptor loci generated from genetic distance in GenALEx v.6.5b3 between (**A**) species (Red-chested Flufftail, White-winged Flufftail [Ethiopia] and White-winged Flufftail [South Africa], where axis 1 and axis 2 explains 74.68% and 8.01% of the variance across species in TLR gene diversity, respectively), and between (**B**) populations (White-winged Flufftail [Ethiopia] and White-winged Flufftail [South Africa], where axis 1 and axis 2 explains 31.66% and 26.88% of the variance across species in TLR gene diversity, respectively). One symbol represents one individual. X = Red-chested Flufftail, Δ = White-winged Flufftail (South Africa) and ◾ = White-winged Flufftail (Ethiopia).

The clustering of the two flufftail species observed in the PCoA (Fig. 1) demonstrates clear separation of the two species using TLR data (percentages of variation explained by the first two axes were 74,68% and 8,01%, respectively). These results indicate that the two different species of flufftail vary in the selective pressure on TLRs due to different evolutionary contraints, life-history traits and environmental effects, resulting in a varying suite of TLR alterations. A population-level PCoA demonstrates an absence of differentiation in the two White-winged Flufftail populations from Ethiopia and South Africa. The variation explained by the first two axes in the population-level PCoA is lower (percentages of variation explained by the first two axes were 31,66% and 26,88%, respectively) than observed for the species level variation.

### Comparison of TLR gene diversity in Flufftail with other bird species

**Figure 2:**
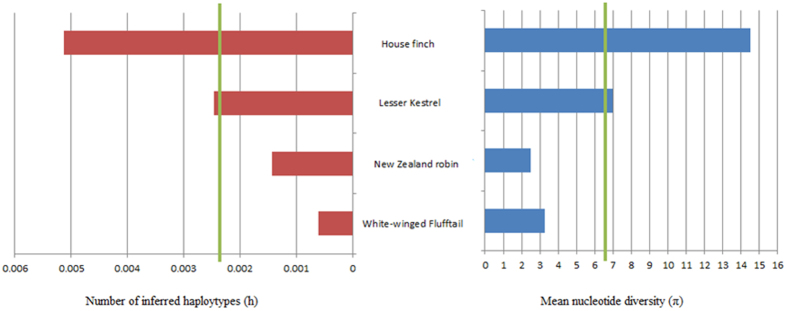
Variance in estimates of the number of inferred haploytypes (h, indicated on the right in blue bars) and mean nucleotide diversity (π, indicated on the left in red bars) among avian species (house finch, Lesser Kestrel, New Zealand Robin and White-winged Flufftail) calculated from three Toll-like receptors (TLR1LB, TLR3 and TLR4). Median values for h and π, respectively, for the total sample set (n = 36) are shown as green vertical lines.

TLR diversity in White-winged Flufftail from Ethiopia (Ho = 0.128; He = 0.145; uHe = 0.157) and South Africa (Ho = 0.190; He = 0.131; uHe = 0.157) was found to be similar. However, TLR diversity in White-winged Flufftail from Ethiopia and South Africa was found to be lower (Ho = 0.159; He = 0.138; uHe = 0.157) in comparison to Red-chested Flufftail (Ho = 0.452; He = 0.399; uHe = 0.479), and New Zealand Robin (*Petrocia austalis rakiura*) (Ho = 0.417; He = 0.477; 17) as shown in Table 3. A comparison between the TLRs of White-winged Flufftail and other bird species of similar sample size (n = 8–10) was conducted based on mean nucleotide diversity (π) and number of inferred haplotypes (*h)*, as shown in Table 4. Analyses included a threatened species that has undergone several population bottlenecks (New Zealand Robin [*Petroica australis rakiura*]), as well as more common species, house finch (*Carpodacus mexicanus*) and Lesser Kestrel (*Falco naumanni*). A comparison of the number of inferred haplotypes and mean nucleotide diversity for three TLRs (TLR1LB, TLR3 and TLR4) in four species, for which data was available, was conducted and is shown in Fig. 2. The number of haplotypes (*h)* ranged from 2 to 20 with a mean *h* = 6.8 and nucleotide diversity ranged from 0 to 0.067 with a mean π = 0.0024. Diversity estimates for White-winged Flufftail and New Zealand Robin were low compared to more common species; house finch and Lesser Kestrel (Fig. 2 and Table 4). Viral TLR diversity of TLR3 and TLR7 in White-winged Flufftail (7 haplotypes) was similar to Lesser Kestrel (6 haplotypes) and higher than New Zealand Robin (3 haplotypes) whereas non-viral TLR diversity (TLR1LA, TLR1LB, TLR4) in White-winged Flufftail (10 haplotypes) was similar to New Zealand Robin (9 haplotypes) and lower than Lesser Kestrel (33 haplotypes).

## Discussion

Our study is the first to examine genetic diversity of TLR genes in the Critically Endangered White-winged Flufftail which has an estimated population size of less than 250 adults remaining globally in the wild. Principal Coordinate Analysis of White-winged Flufftail and Red-chested Flufftail provided evidence of differentiation between the species and is in line with another study of TLR variation across species that reported variation according to the taxonomic groups^25^. A lack of TLR differentiation between two populations (Ethiopia and South Africa) of White-winged Flufftail was observed, providing evidence that these populations are not genetically different at the study set of genes.

A significant deviation from Hardy-Weinberg expectations was observed for TLR1LA in the White-winged Flufftail population from Ethiopia. Deviations from Hardy-Weinberg may be attributed to allelic dropout, a Wahlund Effect, a population history of inbreeding or selection acting at that locus^37^. However, in this case, allele dropout is unlikely due to the high quality of the DNA obtained to sequence the amplicons, the unidirectional production of sequences and the absence of significant deviations in the other populations tested (White-winged Flufftail from South Africa and Red-chested Flufftail). In addition, a Wahlund effect appears unlikely based on the PCoA results. Thus, deviations from Hardy-Weinberg, in this case, may be due to inbreeding or selection. By comparing rates of synonymous (*d*_*S*_) and non-synonymous (*d*_*N*_) substitutions, we found a non-significant (P > 0.05) excess of *d*_*N*_ to *d*_*S*_ in TLR1LA (*d*_*N/*_/*d*_*S*_ = 1.644; Table 2) using the SLAC model, providing evidence of balancing selection. Furthermore, a non-significant excess of synonymous substitutions over non-synonymous alterations was observed in two TLR loci (TLR1LB and TLR7), compatible with purifying selection of the sites, which was further supported by the observation of negative Tajima D values (Table 2). An absence of positive selection acting on amino acid sites was observed for all TLRs investigated, however, at least one negatively selected codon (codon 190) within the TLR7 gene of the White-winged Flufftail was detected (Table 2). Several studies have indicated that TLR sequences are characterized by purifying selection, with specific TLR codons being subjected to positive selection^38^,^39^. An absence of positive selection observed in this study may be influenced by the number of taxa investigated^40^ and more detailed surveys of a larger number of bird species will be required in order to provide a more accurate assessment of positive selection.

Diversity estimate patterns for White-winged Flufftail were lower compared to Red-chested Flufftail, and were comparable to estimates reported for New Zealand Robin (*Petroica australis rakiura*) which included a similar sample size to the study presented here (n = 10). The New Zealand Robin represents a population of birds on Ulva Island (New Zealand) that has undergone severe bottlenecks. The population, although previously common, declined to less than 300 individuals due to predation on Stewart Island and 25 individuals were used to introduce robins to Ulva Island^16^. TLR diversity of White-winged Flufftail was found to be lower compared to two widespread species; house finch (*Carpodacus mexicanus*) and Lesser Kestrel (*Falco naumanni*)^25^. Although direct comparisons between species on TLR diversity are complicated, due to each species showing variation in evolutionary, ecological and life-history traits, population level analysis of TLR diversity for species of conservation concern and common species, provides a starting point for studies on TLR diversity and its effects on fitness and survival. TLR genes play a key role in host defence, and variation within these genes is an important component of adaptive genetic diversity^41^. Thus, high diversity at TLR loci may indicate the potential of a population to adapt to changing environments, and reduced TLR diversity may have an effect on the survival of individuals^41^. In addition, species with high immune diversity at TLR loci may have reduced survival probabilities if maladaptive variations are present^42^. In the present study, the effect of low genetic diversity at TLR loci and how it will relate to future evolutionary potential of White-winged Flufftail populations is unknown. However, low TLR diversity in White-winged Flufftail provides evidence that this species is more likely to be threatened by changes to the environment, including anthropogenic threats such as alterations of the landscape due to agriculture, which would potentially expose the species to new diseases. As a measure to reduce extinction risk of the White-winged Flufftail, an artificial breeding facility using collected eggs may be considered, taking into account that this could result in further genetic depletion due to the creation of an artificial bottleneck. Re-introductions should only be considered for areas where the species has been reported to occur, as translocations of this species into new environments could result in the species being exposed to a novel pathogenic landscape. It should be a priority for conservation efforts to be directed towards maintaining pristine habitat for White-winged Flufftail in its current distribution range. This can be achieved by combating threats (e.g. overgrazing) and opposing developments that can negatively impact the habitat of this specialized species, in both South Africa and Ethiopia. It should ideally result in critical, important sites being declared as formally protected areas. Suitable breeding habitat is already under severe pressure in Ethiopia and the protection thereof should be a priority.

## Materials and Methods

### Samples

Our aim was to collect as many samples from both countries to ensure as much representation of the genetic make-up as possible. Great care was taken while working with and sampling White-winged Flufftail which is a highly threatened bird species. We aimed to ensure that our impacts were limited and the handling time of the birds minimised. Blood samples were collected from the media metatarsal vein by a qualified veterinarian and never exceeded 1% of body volume (*i.e*. 0.3 ml). All birds handled were banded with a standard Safring metal ring by a qualified ringer. Collected blood samples were either stored in Queen’s Lysis buffer or on Whatman FTA^®^ paper. In one case, where a dead bird was found in Franklin Vlei following a powerline collision, the whole bird was stored in 70% ethanol and a tissue sample was collected from the footpad of the bird. White-winged Flufftail (*Sarothrura ayresi*) samples were obtained from South African birds at three different localities namely Wakkerstroom (27°21′25.11″S, 30°07′13.50″E) in January 2014, Franklin Vlei (30°24′03.6″S, 29°27′13.3″E) in March 2001 and Middlepunt (25°32′43.7″S, 30°07′11.3″E) in February 2014. White-winged Flufftail (*Sarothrura ayresi*) samples were obtained from Berga, Ethiopia (9°16′01.2″N, 38°22′58.8″E) in August 2013. Lastly, Red-chested Flufftail (*S. rufa*) samples were obtained from birds caught in Wakkerstroom (27°21′25.11″S, 30°07′′13.50″E) in January 2013. The trapping of White-winged Flufftail in the wild is notoriously difficult and attempts were more successful in Ethiopia. The birds in South Africa were collected through mist netting. Mist nets of 12 m in length were used across the wetlands. In Ethiopia, individual flufftails were located while moving swiftly through the wetland. Such individuals were then targeted with a butterfly net. The success of capturing in these wetlands is due to 1) difference in habitat; the sedges in the seasonal wetland in Ethiopia are shorter and thus easier to move swiftly through, while mobility is more restricted in the dense permanent peat wetlands consisting of Carex-dominated sedges and reedbeds (dominated by tall *Typha* and *Phragmites*) in South Africa. The second reason is due to the difference in the densities of birds – there is only one known breeding site in Ethiopia remaining and the birds occur here in relative high densities during the short breeding season in the boreal summer, whereas the migrating individuals are well spread over a number of wetlands in South Africa during the austral summer. Sampling in Ethiopia took place under the Ethiopian permit no. ET-TH/003/2013 and South African import no. CPC5–1574. In South Africa, sampling was done under the Mpumalanga Tourism and Parks Agency permit no. 2014 MPB 5384. Approval for the project was obtained from the Research Ethics and Scientific Committee of the National Zoological Gardens of South Africa (NZGP15/16) and from the BirdLife South Africa Ethics Committee (2015/04/B), and was carried out in accordance with the approved guidelines.

### Genomic DNA Isolation, Amplification and Sequencing

DNA extraction was conducted using the QIAamp^®^ DNA Investigator kit (Qiagen), according to the manufacturer’s protocol. Primers developed for members of Apterygiformes, Gruiformes, Psittaciformes and Passeriformes (30; Table 5), were used to target portions of nine TLR gene regions, namely TLR1LA, TLR1LB, TLR2, TLR3, TLR4, TLR5, TLR7, TLR15 and TLR21. Amplification was carried out in separate PCR reactions, consisting of 1 × DreamTaq Green PCR Master Mix, 0.4 μM of each primer and approximately 20 ng template DNA, in a total volume of 20 μl. The temperature profile was as follows: an initial denaturation at 95 °C for 3 min, 35 cycles of 95 °C for 30 s, 53–58 °C for 30 s, and 72 °C for 1 min, followed by a final extension at 72 °C for 10 min. Successful PCR products were purified with Exonuclease I and FastAP (Thermo Fisher Scientific Inc.). Gene fragments were sequenced in both directions, using the BigDye Terminator v3.1 Cycle Sequencing Kit and visualised on a 3 500 Genetic Analyser (Applied Biosystems). Sequence chromatograms were edited and assembled using Geneious v.8.0.3 (created by Biomatters).

### Identification of synonymous SNPs

Synonymous and non-synonymous SNP variations were determined by translating the TLR gene nucleotide sequences to the longest open reading frames. The identity and integrity of the respective amino acid sequences were confirmed by standard protein BLAST. Amino acid variations were visually inspected using BioEdit v.7.0.9.0 43.

### Genetic differentiation and diversity

Differences between populations and species in terms of observed heterozygosity (Ho), mean expected heterozygosity (He), unbiased expected heterozygosity (uHe) and Hardy-Weinberg Equilibrium (HWE) were determined using GenALEx 6.5b3 (44). The pattern of allelic differentiation between species and populations was explored via Principal Coordinate Analysis (PCoA) using GenALEx 6.5b3 44. We used DnaSP v5.1 45 to compare the number of polymorphic (SNPs) sites, the number of haplotypes (*h*) and the nucleotide diversity (π) among sequences, Watterson’s estimator of the population mutation rate (θ*w*) and the average number of nucleotide differences between alleles (*k*). In order to survey TLR genetic variation and place White-winged Flufftail variation in the context of sequences from other species in the vertebrate class Aves, we performed a comparison of average π and *h* values obtained from this and published studies (Fig. 2), which were plotted graphically in Microsoft Excel.

### Codon-based analyses of positive selection

Positive selection was inferred using the HyPhy package^46^, implemented in the Data Monkey Web Server ( http://www.datamonkey.org). Positive selection is characterized by an excess of non-synonymous (*d*_*N*_) over synonymous (*d*_*S*_) substitution rates, whereas negative or purifying selection is due to an excess of synonymous substitutions due to constraints in protein structure and biological function. TLR gene sequences were analyzed using the random effects likelihood (REL) model, as well as the more conservative single likelihood ancestral counting (SLAC) method. Statistical significance at *P* < 0.1 and Bayes Factor >50 was set for SLAC and REL analyses, respectively. To determine the presence of significant recombination breakpoints within the TLR genes examined, the respective gene alignments were subjected to GARD analysis^47^, as implemented in the Data Monkey Web Server (http://www.datamonkey.org).

## Additional Information

**How to cite this article**: Dalton, D. L. *et al.* Lack of diversity at innate immunity Toll-like receptor genes in the Critically Endangered White-winged Flufftail (*Sarothrura ayresi*). *Sci. Rep.* **6**, 36757; doi: 10.1038/srep36757 (2016).

**Publisher’s note:** Springer Nature remains neutral with regard to jurisdictional claims in published maps and institutional affiliations.

## Supplementary Material

Supplementary Information

## Figures and Tables

**Table 1:** Polymorphisms in Flufftail Toll-like receptors.

Population	TLR1LA	TLR1LB	TLR3	TLR4	TLR7	Total
Red-chested Flufftail	4 (1)	4 (0)	6 (4)	2 (2)	4 (0)	**20 (7)**
White-winged Flufftail (Ethiopia)	1 (2)	2 (2)	1 (0)	0 (0)	2 (1)	**6 (5)**
White-winged Flufftail (South Africa)	1 (3)	2 (0)	1 (0)	0 (0)	2 (1)	**6 (4)**
White-winged Flufftail (all)	**1 (4)**	**3 (2)**	**1 (0)**	**0 (0)**	**2 (2)**	**7 (8)**

Synonymous SNPs indicated outside of brackets and non-synonymous SNPs in the coding regions indicated in brackets.

**Table 2:** Selection (characterized by non-synonymous (*d*_*N*_) and synonymous (*d*_*S*_) substitution rates) and polymorphism estimates (Watterson’s estimator of the population mutation rate (θ*w*) and the average number of nucleotide differences between alleles (*k*)) for White-winged Flufftail Toll-like receptors (TLR1LA, TLR1LB, TLR3, TLR4 and TLR7).

Locus	Species	Fragment length (aa)	Sites under selection			Polymorphic estimates		
			SLAC	REL	*d*_*N*_*/d*_*S*_	*k*	θ*w*	Tajima’s D
TLR1LA	White-winged Flufftail	214	0	0	1.644	1.267	0.0027	−1.136
TLR1LB	White-winged Flufftail	320	0	0	0.307	1.333	0.0018	−0.985
TLR3	White-winged Flufftail	380	N/A^+^	N/A^+^	N/A^+^	0.467	0.0003	0.820
TLR4	White-winged Flufftail	210	N/A^+^	N/A^+^	N/A^+^	N/A^^^	0.0000	N/A^^^
TLR7	White-winged Flufftail	412	1 (190)	2 (190, 220)	0.274	0.467	0.0011	−0.038

*d*_*N*_/*d*_*S*_ was calculated using the SLAC model implemented in the Data Monkey Web Server; ^+^N/A: indicates that more than three unique sequences for selection not available; ^^^N/A indicated that no polymorphic sites were observed.

**Table 3:** Observed, expected heterozygosity and unbiased heterozygosity estimates for five Toll-like receptor loci genotyped in White-winged Flufftail (Ethiopia and South Africa) and Red-chested Flufftail.

Population	N	Ho	He	uHe
Red-chested Flufftail	**3**	**0.452**	**0.399**	**0.479**
White-winged Flufftail (Ethiopia)	7	0.128	0.145	0.157
White-winged Flufftail (South Africa)	3	0.190	0.131	0.157
White-winged Flufftail (All)	**10**	**0.159**	**0.138**	**0.157**

N: Number of samples; Ho: mean observed heterozygosity; He: mean expected heterozygosity and uHe: unbiased expected heterozygosity.

**Table 4:** Comparison of Toll-like receptor alterations and diversity measures between White-winged Flufftail, New Zealand Robin, Lesser Kestrel and house finch.

Locus	Species	n	SNPs	*h*	π	Reference
TLR1LA	White-winged Flufftail	10	5	4	0.0020	This study
	New Zealand Robin	10	2	2	0.0009	Grueber *et al.*, 2012
	Lesser Kestrel	8	19	11	0.0039	Alcaide and Edwards, 2011
	house finch	51	44	62	0.0058	Alcaide and Edwards, 2011
TLR1LB	White-winged Flufftail	10	5	5	0.0014	This study
	New Zealand Robin	10	3	2	0.0016	Grueber *et al.*, 2012
	Lesser Kestrel	8	16	15	0.0039	Alcaide and Edwards, 2011
	house finch	8	25	20	0.0067	Alcaide and Edwards, 2011
TLR3	White-winged Flufftail	10	1	2	0.0004	This study
	New Zealand Robin	9	0	1	0.0000	Grueber *et al.*, 2012
	Lesser Kestrel	8	1	2	0.0009	Alcaide and Edwards, 2011
	house finch	8	11	9	0.0038	Alcaide and Edwards, 2011
TLR4	White-winged Flufftail	10	0	1	0.0000	This study
	New Zealand Robin	10	4	5	0.0027	Grueber *et al.*, 2012
	Lesser Kestrel	8	6	7	0.0026	Alcaide and Edwards, 2011
	house finch	8	16	14	0.0049	Alcaide and Edwards, 2011
TLR7	White-winged Flufftail	10	4	5	0.0011	This study
	New Zealand Robin	10	3	≥ 2	N/A	Grueber *et al.*, 2012
	Lesser Kestrel	8	3	4	0.0017	Alcaide and Edwards, 2011
	house finch	8	27	15	0.0077	Alcaide and Edwards, 2011

n: number of samples; SNPs: number of SNPs detected; *h*: the number of inferred haplotypes; π: mean nucleotide diversity

**Table 5:** PCR primers used for amplification of six TLR genes in Flufftails.

Genes	Fragment Length (bp)	F/R	Primer Sequence 5’−3’	Target locus	T_a_
TLR1LA	644	F	GATGGAATGAGCACTTCAGA	Exon 2	58 °C
		R	CTTCGTCTGCGTCACTG		
TLR1LB	962	F	TCCAGGYTWCAAAATCTGACAC	Exon 1	55 °C
		R	CGGCACRTCCARGTAGATG		
TLR3	1141	F	CAAWGTTGAACTTGGTGAAAAT	Exon 4	53 °C
		R	TCACAGGTRCAATCAAANGG		
TLR4	631	F	GAGACCTTGATGCCCTGAG	Exon 3	55 °C
		R	CCATCTTRAGCACTTGCAAAG		
TLR5	749	F	CCAAATGCCCAAATCCTTTC	Exon 1	51 °C
		R	GTGGGAAAAGCCCAGGAG		
TLR7	1237	F	GTATCTKGGACARAACTGYTA	Exon 2	55 °C
		R	TYGAAGAGATTGGCTTTCC		
